# Immunostimulatory Activity of a Mixture of *Platycodon grandiflorum*, *Pyrus serotine*, *Chaenomeles sinensis*, and *Raphanus sativus* in RAW264.7 Macrophages

**DOI:** 10.3390/ijms251910660

**Published:** 2024-10-03

**Authors:** Weerawan Rod-in, Minji Kim, A-yeong Jang, Yu Suk Nam, Tae Young Yoo, Woo Jung Park

**Affiliations:** 1 Department of Marine Bio Food Science, Gangneung-Wonju National University, Gangneung 25457, Gangwon, Republic of Korea; weerawanro@nu.ac.th (W.R.-i.); jay941006@gmail.com (A.-y.J.); 2 Department of Agricultural Science, Faculty of Agriculture Natural Resources and Environment, Naresuan University, Phitsanulok 65000, Thailand; 3 Center of Excellence in Research for Agricultural Biotechnology, Naresuan University, Phitsanulok 65000, Thailand; 4 Department of Wellness-Bio Industry, Gangneung-Wonju National University, Gangneung 25457, Gangwon, Republic of Korea; mjikim9731@gmail.com; 5 Department of Food Science and Technology, Gangneung-Wonju National University, Gangneung 25457, Gangwon, Republic of Korea; 6 NAAAMYUUU FNC Co., Ltd., 20, Juheung, Seocho, Seoul 06540, Republic of Korea; kjoslove@naaamyuuu.com; 7 FD FARM Co., Ltd., Icheon 17300, Gyeonggi, Republic of Korea; fd-farm@fd-farm.com; 8 KBIoRANCh Co., Ltd., Gangneung 25457, Gangwon, Republic of Korea

**Keywords:** inflammation, macrophages, cytokines, immunomodulatory, *Platycodon grandiflorum*

## Abstract

In this study, a mixture of *Platycodon grandiflorum*, *Pyrus serotina*, *Chaenomeles sinensis*, and *Raphanus sativus* (PPCRE) was investigated for their immuno-enhancing effects, as well as the molecular mechanism of PPCRE in RAW264.7 cells. PPCRE dramatically increased nitric oxide (NO) and prostaglandin E_2_ (PGE_2_) generation depending on the concentration while exhibiting no cytotoxicity. PPCRE markedly upregulated the mRNA and protein expression of immune-related cytotoxic factors such as cyclooxygenase (COX)-1, COX-2, and inducible nitric oxide synthase (iNOS) and pro-inflammatory cytokines such as interleukin (IL)-1β, IL-6, and tumor necrosis factor alpha (TNF-α), as well as the mRNA level of *IL-4*. PPCRE increased the mitogen-activated protein kinase (MAPK) signaling pathway by upregulating the phosphorylation of extracellular signal-regulated kinase (ERK), stress-activated protein kinase/Jun N-terminal-kinase (SAPK/JNK), and p38. Furthermore, PPCRE considerably activated the nuclear factor kappa B (NF-κB) signaling pathway by increasing phosphorylation of NF-κB-p65. PPCRE-stimulated RAW264.7 cells increased macrophage phagocytic capacity. In conclusion, our study found that PPCRE improved immune function by modulating inflammatory mediators and regulating the MAPK and NF-κB pathway of signaling in macrophages.

## 1. Introduction

Immunostimulants are generally substances that have the potential to increase non-specific immune responses by activating neutrophils and macrophages [1]. The macrophage serves an essential function in triggering both the adaptive and innate immune systems and produces immune mediators such as nitric oxide (NO), prostaglandins (PGs), and cytokines, all of which contribute to immunological cascades [2,3,4]. Recently, natural products or foods have been reported to modulate immune responses [5,6], and several studies have shown that mixing substances effectively improves synergetic the immune-enhancing effects [7,8,9,10,11,12].

*Platycodon grandiflorum*, often known as balloon flower, represents a clump-forming perennial plant from the Campanulaceae family that has been presented to contain multiple chemical substances, notably, platycodin (D, D3, E, G3, and N), platycoside (A, D, E, and O), polysaccharides, quercetin, luteolin 7-*O*-glucoside, apigenin, phenolic acids, caffeic acid, *p*-coumaric, and fatty acids [13]. *P. grandiflorum*, a traditional botanical medicine, is used extensively in Northeast Asia (China, Japan, and Korea) to cure coughs, excessive phlegm, and sore throats [13]. In Korea, *P. grandiflorum* is used to treat various respiratory disorders and is also used as a Korean food ingredient [14]. A variety of bioactive effects have been found in *P. grandiflorum* extracts, including antioxidant, anti-obesity, anti-inflammatory, immunomodulatory, anti-atherosclerotic, and anticancer properties [15,16,17,18,19]. Moreover, they also activate immune-enhancing effects in both in vitro and in vivo systems [20,21,22], and the extracts of *P. grandiflorum* combined with many plants have shown anti-obesity, anti-inflammatory, and vasodilatory effects [23,24].

*Chaenomeles sinensis* is herbal medicine of the Rosaceae family and is cultivated in Japan, Korea, China, Bhutan, and Burma [25]. *C. sinensis* is rich in essential oils, triterpenes, and phenolic compounds consisting of quercetin, flavonoids, catechins, and procyanidins, and it has been shown that *C. sinensis* possesses anti-influenza, antioxidant, anti-aging, and anti-inflammatory effects [25,26,27]. A mixture of *C. sinensis*, *Aralia elata*, and *Glycyrrhizae radix* inhibited neuroprotective efficacy regarding ischemia-induced damage to the brain in rats [26]. One of the mixed plant extracts, Asian pear (*Pyrus serotina* Rehd., *Py. serotina*), is a popular horticultural plant within Japan, China, and Korea of the Rosaceae family [27]. The pear has been utilized as a sweet fruit and dietary supplement, as well as in folk medicine to alleviate fever, suppress coughs, quench thirst, relieve pain, and prevent tissue decomposition in internal burns, due to its potent active compounds such as phenolic compounds (phenolic acids and flavonoids), triterpenes, and glucosides [28]. Lastly, Radish (*Raphanus sativus* L.), which belongs to the Brassicaceae family, is one of the most widely consumed foods in the world and is used as herbal medicine. It has been previously found to contain anticancer, antiviral, anti-inflammatory, and immunostimulatory properties [29,30,31].

In our preliminary research, we discovered that the combination of *P. grandiflorum* and *Salvia plebeia* exhibited immune-enhancing effects in RAW264.7 cells [32]. Each compound has been studied and known to be important for human health; however, no study has reported that the mixture of the compounds has also been important and provides an immunostimulatory effect. Numerous studies have shown that combining different plant species can boost immune function more effectively than using a single species alone [7,12]. Additionally, there has been evidence of the biological activities of individual plant extracts alone from *P. grandiflorum* [17,20], *Py*. *serotina* [33], *C. sinensis* [25,34], and *R. sativus* [31], but no investigations exist to investigate the impact of the *P. grandiflorum*, *Py*. *serotina*, *C. sinensis*, and *R. sativus* combination (PPCRE) on immune responses. Consequently, the primary objective of the current investigation was to assess the immune-enhancing effects caused by PPCRE and its mechanism of action in mouse macrophages.

## 2. Results

### 2.1. Identification of PPCRE by Ultra-Performance Liquid Chromatography–Mass Spectrometry/MS (UPLC-MS/MS) Analysis

**Figure 1:**
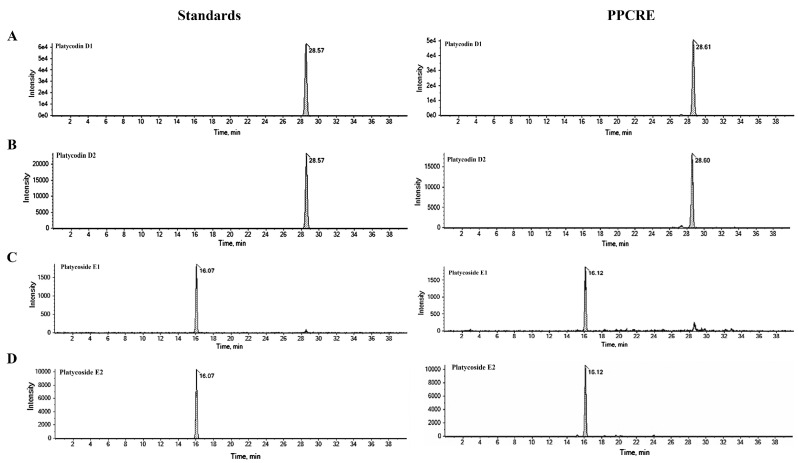
UPLC-MS/MS analysis of PPCRE. (**A**) platycodin D1, (**B**) platycodin D2, (**C**) platycoside E1, and (**D**) platycoside E2. The left side shows HPLC chromatograms at the retention times and peaks of a standard solution. The right side shows HPLC chromatograms at the retention times and peaks of PPCRE.

The UPLC-MS/MS analysis identified the PPCRE that contained four saponins, including platycodin D1, platycodin D2, platycoside E1, and platycoside E2. The chromatograms using standard indicator compounds and PPCRE are shown in Figure 1. According to the assessment of retention times to standards, four metabolites of the major components of PPCRE, including platycodin D1, platycodin D2, platycoside E1, and platycoside E2, were identified in the peak chromatogram of PPCRE. Platycoside E1 and platycoside E2 were detected at 16.12 and 16.12 min, respectively. Platycodin D1 and platycodin D2 were detected at 28.61 and 28.60 min, respectively. In addition, the content of four components in PPCRE were calculated as follows: 3.00 ± 0.19 mg/g of platycodin D1, 2.82 ± 0.18 mg/g of platycodin D2, 0.22 ± 0.02 mg/g of platycoside E1, and 0.34 ± 0.01 mg/g of platycoside E2. Thus, chromatographic analysis confirmed the presence of saponin indicator components in PPCRE.

### 2.2. Cytotoxicity of PPCRE on RAW264.7 Macrophages

**Figure 2:**
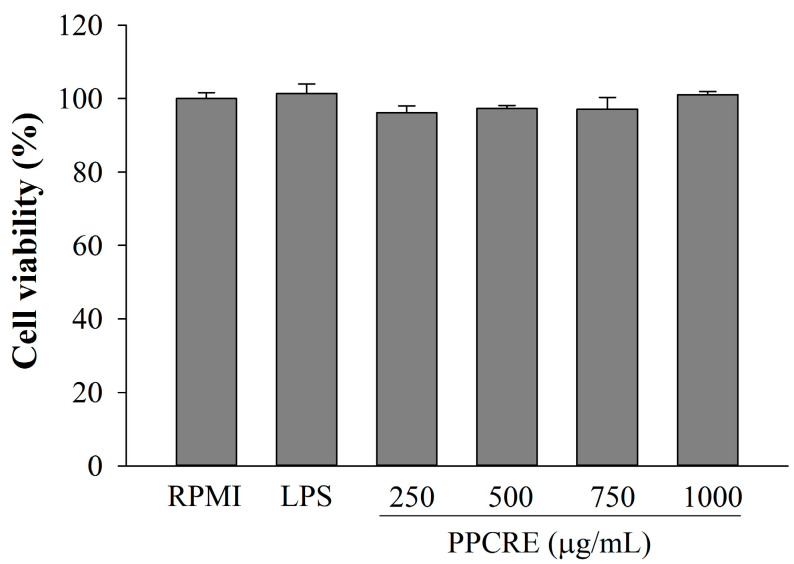
Cytotoxic effects of PPCRE on RAW264.7 macrophages. The data represent three separate experiments and are presented as the mean ± SD (*n* = 3). Compared with RPMI (untreated cells).

After incubating with varying dosages of PPCRE for 24 h, WST assays were utilized to assess the viability of RAW264.7 cells treated with PPCRE. Figure 2 shows that PPCRE was actually non-toxic to RAW264.7 cells at 250–1000 µg/mL, with a statistically significant difference from the untreated cells (RPMI).

### 2.3. Enhancement of NO Production and iNOS mRNA Expression of PPCRE

**Figure 3:**
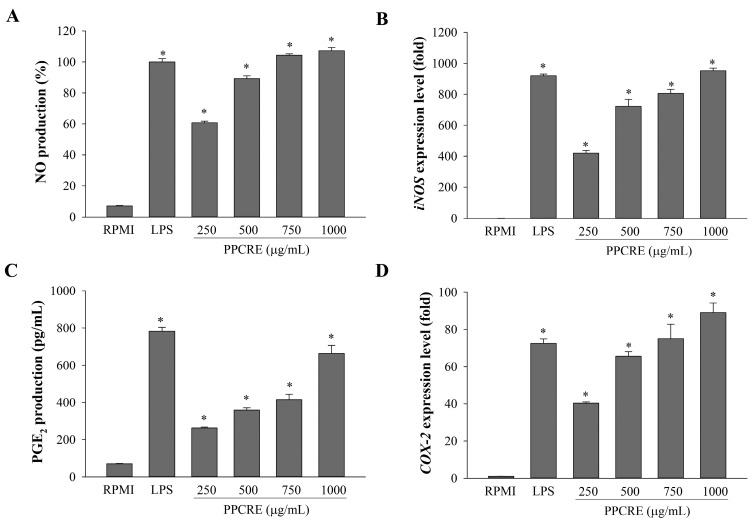
Effects of PPCRE on production and expression of inflammatory mediators in RAW264.7 cells. The supernatants were assayed for the production of NO (**A**) and PGE_2_ (**B**) using the Griess assay and ELISA kit, respectively. The relative mRNA levels of *iNOS* (**C**) and *COX-2* (**D**) were analyzed by qRT-PCR. The data represent three separate experiments and are presented as the mean ± SD (*n* = 3). Compared with RPMI (untreated cells), * *p* < 0.05.

As shown in Figure 3A, NO production dramatically increased in RAW264.7 cells after stimulation with lipopolysaccharides (LPS). Treatment with PPCRE (250–1000 µg/mL) demonstrated a statistically significant dose-dependent increase in NO production, and the high concentrations of PPCRE also showed higher NO contents than LPS. Similarly, the mRNA expression of inducible nitric oxide synthase (iNOS) was also upregulated by PPCRE dose-dependently (Figure 3B), indicating that PPCRE modulated NO production by regulating *iNOS* expression.

### 2.4. PPCRE Induces PGE_2_ Production and COX-2 mRNA Expression

In response to inflammation, macrophages create prostaglandin E_2_ (PGE_2_) via the metabolic conversion of arachidonic acid by cyclooxygenase (COX)-2 [1]. To assess the effect of PPCRE on PGE_2_ production in RAW264.7 cells, the concentrations of PGE_2_ in the cultivated supernatants were measured by an ELISA assay. The production of PGE_2_ was observed in a dose-dependent manner (Figure 3C). It was found that PPCRE (250, 500, 750, and 1000 µg/mL) significantly elevated PGE_2_ production by 34%, 46%, 53%, and 85%, respectively. Following LPS treatment, PGE_2_ production was significantly higher than with RPMI.

To further demonstrate whether PPCRE influenced the transcription of *COX-2* mRNA, PPCRE or LPS-treated cells were evaluated for *COX-2* expression using real-time qPCR. Figure 3D shows the levels of *COX-2* mRNA that are dose-dependently increased by PPCRE (250–1000 µg/mL). PPCRE at 1000 µg/mL significantly promoted *COX-2* expression in macrophages at a hgher level than with LPS-only treatment. Thus, the results indicated that PPCRE might significantly induce PGE_2_ through the activation of *COX-2* transcription.

### 2.5. PPCRE Enhances Protein Expression Levels of iNOS, COX-1, and COX-2

**Figure 4:**
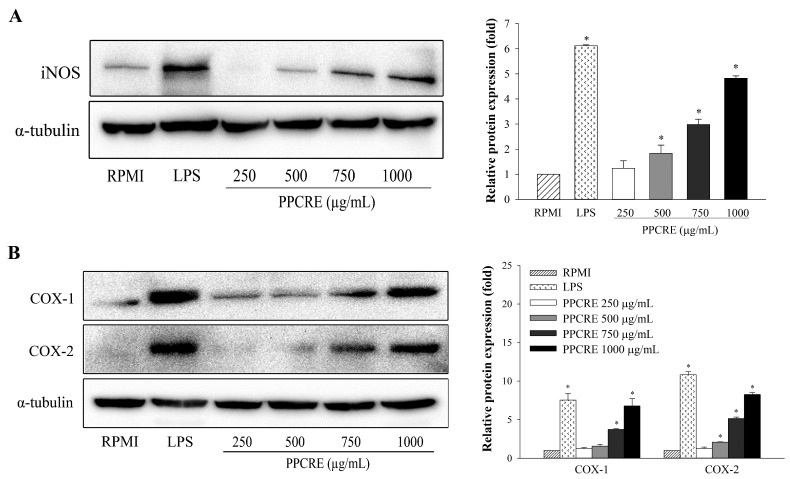
Effects of PPCRE on the protein expression of iNOS, COX-1, and COX-2 in RAW264.7 cells. The relative expression of iNOS (**A**) and COXs (**B**) was analyzed by Western blotting. The data represent three separate experiments and are presented as the mean ± SD (*n* = 3). Compared with RPMI (untreated cells), * *p* < 0.05.

An assay using Western blotting was performed on iNOS, COX-1, and COX-2, which are essential enzymes responsible for producing inflammatory mediators [2,4]. The protein expressions of iNOS and COXs were detected in LPS-activated cells. As displayed in Figure 4, the proteins of iNOS, COX-1, and COX-2 were barely detectable in unstimulated cells, but these were substantially elevated after LPS treatment. PPCRE significantly and concentration-dependently modulated the protein expression of iNOS in macrophages (Figure 4A). PPCRE markedly increased expression of COX-1 and COX-2 protein depending on the concentration (Figure 4B).

### 2.6. PPCRE Enhances the Secretion of Pro-Inflammatory Cytokines

**Figure 5:**
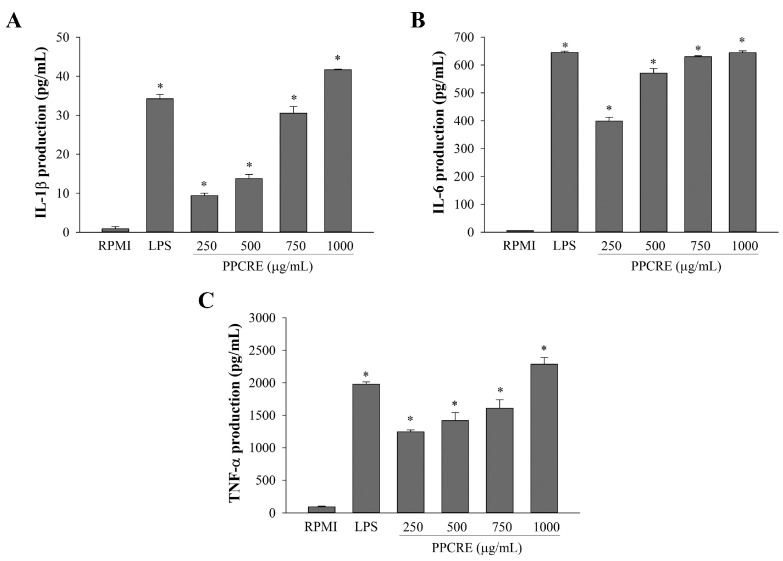
Effects of PPCRE on IL-1β, IL-6, and TNF-α production in RAW264.7 cells. The cytokines IL-1β (**A**), IL-6 (**B**), and TNF-α (**C**) were measured in the medium using an ELISA kit. The data represent three separate experiments and are presented as the mean ± SD (*n* = 3). Compared with RPMI (untreated cells), * *p* < 0.05.

Cytokines are actually intercellular protein signals that possess crucial functions in controlling the function of both innate and adaptive immunity, as previously reported [3,4,10]. The effect of PPCRE on the release of pro-inflammatory cytokines such as interleukin-1β, (IL-1β), IL-6, and tumor necrosis factor-α (TNF-α) in RAW264.7 cells was studied using an ELISA assay. As shown in Figure 5, LPS treatment considerably boosted the production of three kinds of cytokines compared to RPMI. PPCRE was found to significantly increase cellular release of IL-1β, IL-6, and TNF-α. PPCRE (250–1000 µg/mL) increased IL-1β production by 27–122% (Figure 5A), IL-6 production by 62–100% (Figure 5B), and TNF-α production by 63–115% (Figure 5C). Furthermore, PPCRE at 1000 µg/mL increased IL-1β and TNF-α production significantly more than LPS-only treatment, while IL-6 production was comparable.

**Figure 6:**
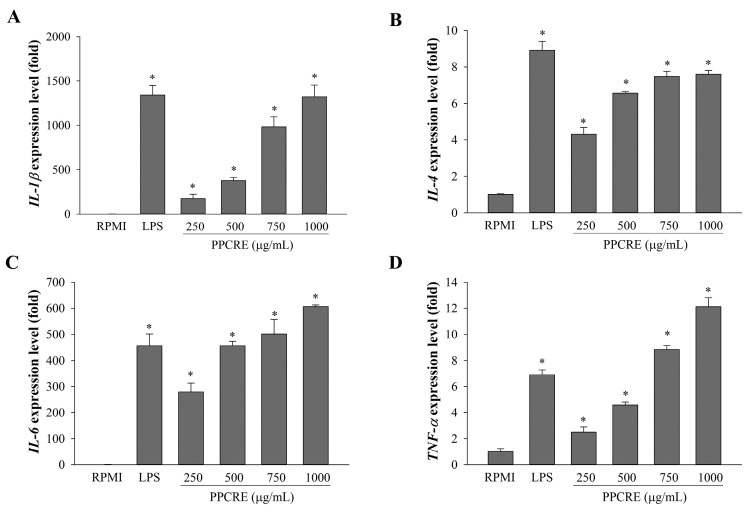
Effects of PPCRE on the cytokine expression in RAW264.7 cells. Real-time qPCR was used to examine the relative mRNA expression of *IL-1β* (**A**), *IL-4* (**B**), *IL-6* (**C**), and *TNF-α* (**D**). The data represent three separate experiments and are presented as the mean ± SD (*n* = 3). Compared with RPMI (untreated cells), * *p* < 0.05.

In addition, the current study examined mRNA levels of four cytokines (*IL-1β*, *IL-4*, *IL-6*, and *TNF-α*) to determine whether PPCRE influenced the enhanced immune effect. PPCRE stimulated *IL-1β*, *IL-4*, *IL-6*, and *TNF-α* expression in a dose-dependent manner (Figure 6A–D). PPCRE had a significant effect on all cytokines when compared to RPMI.

### 2.7. PPCRE Enhances MAPK and NF-κB Activation in RAW264.7 Cells

**Figure 7:**
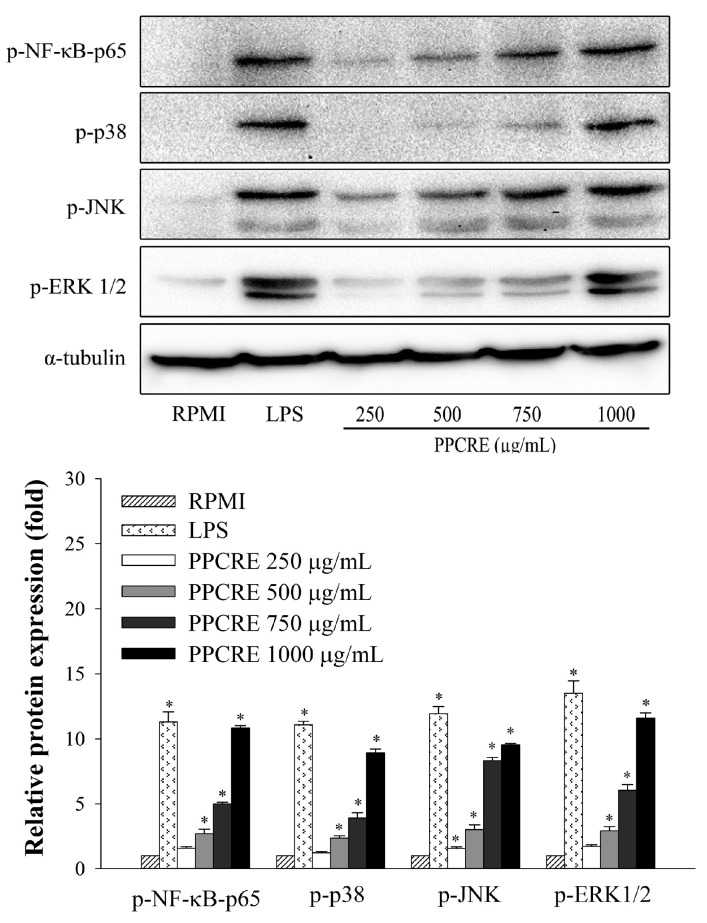
Effect of PPCRE-regulated NF-κB and MAPK phosphorylation. The data represent three separate experiments and are presented as the mean ± SD (*n* = 3). Compared with RPMI (untreated cells), * *p* < 0.05.

To assess the impact of PPCRE on NF-κB and MAPK activation, an immunoblotting assay was used for evaluating the phosphorylation quantities of the NF-κB p65 subunit, extracellular signal-regulated kinase (ERK), p38, and c-Jun NH2-terminal kinase (JNK). As shown in Figure 7, LPS also significantly enhanced the NF-κB-p65 expression levels. Treatment with PPCRE (250–1000 µg/mL) dramatically raised the expression of phosphorylated NF-κB-p65, indicating that PPCRE enhanced immunity by triggering inducing macrophage activation via the NF-κB signaling pathway. A dose-dependent increase in ERK1/2, JNK, and p38 phosphorylation was observed in cells treated with 250–1000 µg/mL PPCRE (Figure 7). In addition, RAW264.7 cells had considerably higher levels of ERK1/2, JNK, and p38 phosphorylation after exposure to LPS alone.

### 2.8. Effects of PPCRE on the Phagocytic Uptake of Macrophages

**Figure 8:**
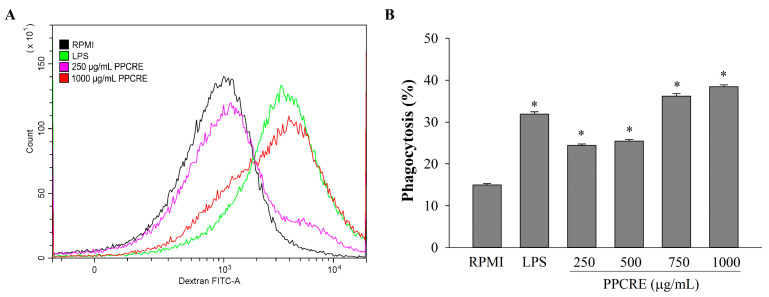
Effect of PPCRE on phagocytic activity. The flow cytometry results showing phagocytosis with FITC–dextran on macrophages after treatment with control and PPCRE at the low and high concentrations (**A**). Representative histograms of PPCRE demonstrating its percentages on viable RAW264.7 cells (**B**). The data represent three separate experiments and are presented as the mean ± SD (*n* = 3). Compared with RPMI (untreated cells), * *p* < 0.05.

To further investigate the possible role of phagocytosis in the effect of PPCRE on RAW264.7 cells, the fluorescence intensity of phagocytes can be assessed through a flow cytometer (Figure 8). PPCRE (250–1000 μg/mL) substantially boosted phagocytotic efficiency compared to RPMI at all doses, and also dose-dependently increased phagocytosis. In addition, the PPCRE demonstrated the greatest increase in fluorescence intensity over LPS at 750 and 1000 μg/mL.

## 3. Discussion

Immunomodulatory agents derived from natural products stimulate immune cells in the body, triggering an immune response to infections [35]. Previous investigations demonstrated that a combination of plant extracts has anti-nociceptive, anti-inflammatory, anti-obesity, anti-malarial, and anticancer properties [23,36,37], especially immune-enhancing activity in macrophages [7,10]. Saponins, as secondary metabolites, are glycosides of triterpenes and steroids, which are one of the majority of prevalent and varied categories of plant-based substances [38]. Among the diverse saponins, platycodin D and platycoside E are the most physiologically relevant and important indicators of *P. grandiflorum* saponins that exhibit immunological effects in immune cells [39,40]. Firstly, we determined the composition of PPCRE by UPLC-MS/MS analysis, and our results found that PPCRE was identified as platycodin D1, platycodin D2, platycoside E1, and platycoside E2, indicating PPCRE might be effective for immune-enhancing effects. Similarly, Noh et al. reported that *P. grandiflorum* extracts contained platycodin D, which was detected by LC-MS/MS [22]. Hence, the primary objective of this study was to assess immune-enhancing activity of PPCRE on RAW264.7 macrophages.

Numerous immunomodulators, including NO, iNOS, COX-2, and pro-inflammatory cytokines, are produced in response to activated macrophages [3,4,10]. NO, as a highly reactive molecule, plays an important signaling role that protects against infectious organisms in the immune system, which is created by inducible nitric oxide synthase (iNOS) [1,41]. In prior reports, a combination of *Astragalus membranaceus*, *Angelica gigas*, and *Trichosanthes kirilowii* significantly increased NO production by enhancing the mRNA and protein levels of iNOS expression in macrophages [10]; likewise, our results suggested that PPCRE could act as an immunostimulant by modulating the NO secretion and *iNOS* expression. COX is an important enzyme in prostaglandin biosynthesis that has two isoforms: COX-1 and COX-2. The COX-1 enzyme is a constitutive enzyme that appears to be engaged in housekeeping and physiological processes in most tissues, whereas the COX-2 enzyme is primarily expressed in immune cells and is able to be activated by many inflammatory stimuli [42]. Our findings showed that PPCRE could promote the expression of iNOS and COX proteins in RAW264.7 cells. Likewise, the extract of *Abelmoschus esculentus* stimulated immune function by raising NO and PGE_2_ production by enhancing the expression of *iNOS* and *COX-2* [4].

Additionally, numerous cytokines, such as IL-1β, IL-4, IL-6, IL-8, IL-10, TNF-α, IFN-γ, and TGF-β, were also produced by activated macrophages [43]. Several plant extracts dramatically boosted mRNA expression levels of these cytokines [6,44]. Subsequently, PPCRE activated macrophages by boosting the release of cytokines such as *IL-1β*, *IL-6*, and *TNF-α*; increasing mRNA expression of those genes; and releasing *IL-4* levels, thereby strengthening immunomodulatory activity. A mixture of *Sasa quelpaertensis* and *Ficus erecta* was found to enhance the production of NO and cytokine production in RAW264.7 cells [7]. Park et al. (2020) studied and reported that fermented *P. grandiflorum* extract could improve immunomodulatory effects in RAW264.7 cells by increasing IL-1β, IL-6, TNF-α, and NO production [21]. Therefore, our findings suggested that PPCRE enhanced immune function by increasing cytokines that stimulate immunity.

Plant extracts can regulate the immune-enhancing effects of pro-inflammatory mediators in activated macrophages of MAPK and NF-κB [1,4,6]. NF-κB acts as a transcription factor that regulates immunological and inflammatory responses by activating macrophages and inducing other genes [45]. Additionally, it is known that the MAPK signaling pathway causes immune cells to activate the immunomodulators such as iNOS, TNF-α, IL-6, and IL-1β, which contributes to the inflammatory response [6,44]. PPCRE could effectively activate macrophages, implying that PPCRE significantly increased phosphorylation levels of NF-κB p65, JNK, ERK, and p38. Bioactive components in plant extracts can influence various signaling pathways, such as NF-κB and MAPK, which play a role in immune cell activation and phagocytosis regulation. These findings revealed that PPCRE affected inflammatory responses via modulating downstream the MAPK and NF-κB pathways.

Phagocytosis, which is produced by macrophages, neutrophils, and monocytes, is a fundamental and important procedure for removing pathogens, foreign particles, and cell debris from the body [3,46]. Plant extracts can enhance phagocytosis due to various bioactive compounds that influence immune system functions. PPCRE markedly increased the phagocytic activity of RAW264.7 cells. A similar study indicated that the exopolysaccharide from *Paecilomyces lilacinus* exhibited a dose-dependent increase in the phagocytic uptake of FITC-dextran up to 600 μg/mL in macrophage cells [2]. The extracts of *P. grandiflorum* and *Kalopanax pictus* increased the ability of macrophages to participate in immunological activities [3,17]. These results concluded that PPCRE might act as an immunomodulator.

## 4. Materials and Methods

### 4.1. Chemicals and Reagents

Standards of platycodin D (CAS: 58479-68-8) and Platycoside E (CAS: 237068-41-6) were purchased from Wuhan ChemFaces Biochemical Co. Ltd. (Wuhan, China). LPS from *Escherichia coli* 055: B5 was purchased from Sigma-Aldrich (St. Louis, MO, USA). RPMI-1640 medium was purchased from Gibco^TM^ (Waltham, MA, USA). Fetal bovine serum (FBS) and penicillin/streptomycin were purchased from Welgene Inc. (Gyeongsan, Republic of Korea). ELISA kits for TNF-α, IL-1β, and IL-6 were purchased from Abcam (Cambridge, UK). The PGE_2_ ELISA kit was procured from Enzo Life Sciences Co., Ltd. (Farmingdale, NY, USA). Anti-iNOS was obtained from Invitrogen (Carlsbad, CA, USA). Anti-COX-2, phospho-nuclear NF-κB-p65, phospho-p38, phospho-ERK1/2, and phospho-JNK were obtained from Cell Signaling Technology (Danvers, MA, USA). Anti-COX-1 and α-tubulin were obtained from Abcam (Cambridge, UK). Goat anti-rabbit IgG (H + L)-HRP was purchased from GenDEPOT (Katy, TX, USA).

### 4.2. Preparation of PPCRE

The PPCRE is a mixture of dried *Py. serotina* (59.5%), *P. grandiflorum* (30.0%), *C. sinensis* (10.0%), and *R. sativus* (0.5%), provided by FD FARM Co., Ltd. (Icheon, Republic of Korea) after manufacturing. Enzyme powder was quantified at 0.05 to 0.1% (*w*/*v*) in purified water. A mixture of enzymes, including Arazyme, HY-8 xylanase, and HY-13 mannanase (Insectbiotech Co., Ltd., Daejeon, Republic of Korea), was prepared in a 1:1:1 (*w*/*w*) ratio. A multi-enzyme supplement with arazyme was used to supplement food and accelerate the breakdown of proteins or other compounds in plant cells [47]. The enzyme powders were added to a portion of the quantified purified water and dissolved until transparent. The remaining purified water was then added to the dissolved enzyme solution, homogenized, and heated to 40 ± 2 °C. Enzyme treatment was performed for 8 h ± 30 min in a stationary state. The extract from the raw material/enzyme solution was obtained by refluxing the sample for 6–8 h at 100 ± 5 °C to speed up the breakdown of certain compounds and to release more internal components of plants. After extraction, PPCRE was concentrated using a vacuum concentrator to a solid content with a Brix of 45 ± 1.0. The yield was 400 mg/g of dry substrate after extraction and concentration. PPCRE was stored at −4 °C until use and then dissolved in sterile water for treatment with cells.

### 4.3. Analysis of Active Compounds by UPLC-MS/MS

The UPLC-MS/MS analyses were carried out on a Capcell Pak C18 UG 120 column, (5.0 μm, 4.6 × 250 mm) by a gradient solvent system of 0.1% formic acid in DW (A) and acetonitrile (B). The gradient consisted of 82% A/18% B in 0 min, 75% A/25% B in 15 min, 70% A/30% B in 30 min, 82% A/18% B in 35 min, and 82% A/18% B in 40 min. The standards and samples were injected at 5 μL, and the flow rate was 1.0 mL/min. The mass spectrometer was monitored in ESI negative mode over the 50–1200 *m*/*z* mass range. The UPLC-MS/MS method for identifying and quantifying platycodin D and platycoside E in PPCRE is described in Tables S1 and S2.

### 4.4. Cell Culture

Murine macrophage cell lines, RAW264.7, were purchased from the Korean Cell Line Bank (Korean Cell Line Research Foundation, Seoul, Republic of Korea) and cultivated in humidified incubators with 5% CO_2_ in RPMI-1640 medium supplemented with 10% fetal bovine serum (FBS) and 1% penicillin/streptomycin (Welgene, Republic of Korea).

### 4.5. Cell Viability Analysis

Cells were seeded in 96-well plates at 1 × 10^5^ cells/well for 24 h. Cells were incubated for another 24 h in RPMI 1640 medium supplemented with 1% FBS and 1% penicillin/streptomycin, with or without PPCRE (250, 500, 750, and 1000 µg/mL) or LPS (1 µg/mL). LPS was used as a positive control. The cytotoxicity of PPCRE was evaluated by the EZ-Cytox Cell Viability Assay Kit (Daeil Labservice, Seoul, Republic of Korea). The culture medium was then removed, and WST solution was added. After 1 h of incubation, the absorbance was detected at 450 nm using a microplate reader (Agilent BioTek, Santa Clara, CA, USA).

$$\mathrm{Cell}\;\mathrm{viability}\;\left(\%\right)=\frac{{\mathrm{The}\;\mathrm{absorbance}\;}_{(\mathrm{OD}\;450\;\mathrm{nm})\;}\mathrm{of}\;\mathrm{treated}\;\mathrm{cells}\;}{{\mathrm{The}\;\mathrm{absorbance}\;}_{(\mathrm{OD}\;450\;\mathrm{nm})\;}\mathrm{of}\;\mathrm{untreated}\;\mathrm{cells}}\;\times \;100$$

### 4.6. Assay of NO and PGE_2_ Production

RAW264.7 cells were plated at a density of 1 × 10^5^ cells/well. The cells were treated with PPCRE (250–1000 µg/mL) or LPS (1 µg/mL), followed by incubation for another 24 h. The concentration of PPCRE-treated supernatants on NO and PGE_2_ production was determined by analyzing the NO level with Griess reagent (Promega, Madison, WI, USA) and the PGE_2_ level with a PGE_2_ ELISA kit (Enzo Life Sciences, Farmingdale, NY, USA), according to the manufacturer’s protocol.

### 4.7. Cytokine Assay

The supernatants of PPCRE-treated cells were centrifuged at 1000× *g* for 20 min, and the supernatants were collected. Following the manufacturer’s instructions, ELISA kits for mouse IL-1β, IL-6, and TNF-α (Abcam, Cambridge, UK) were used to test the cytokine levels in cell supernatants.

### 4.8. qRT-PCR Analysis

The cells used for the qRT-PCR analysis were treated with various doses of PPCRE or LPS for 24 h. Total RNA was prepared using Tri reagent^®^ (Molecular Research Center, Inc., Cincinnati, OH, USA). The High-Capacity cDNA Reverse Transcription Kit (Applied Biosystems, Waltham, MA, USA) was used to reverse transcribe the total RNA to cDNA. Real-time qPCR analysis was conducted on a QuantStudio™ 3 FlexReal-Time PCR System (Thermo Fisher Scientific, Waltham, MA, USA). The reactions were mixed with 2 µL of cDNA (5 ng), 10 µL of TB Green^®^ Premix Ex Taq™ II (Takara Bio Inc., Kusatsu, Japan), 0.4 µL of ROX reference dye (50X), 0.8 µL of forward primer (7.5 μM), and 0.8 µL of reverse primer (7.5 μM). Target gene expression levels were normalized to *β-actin*. The primer sequences used are shown in Table S3.

### 4.9. Phagocytic Uptake Assays

The PPCRE-treated cells were incubated with 1 mg/mL of FITC-dextran (Sigma-Aldrich, St. Louis, MO, USA) for 1 h at 37 °C. After incubation, ice-cold PBS buffer was added, centrifuged, and washed three times. Cells were resuspended in FACS buffer containing 1 × PBS buffer and 2% FBS and were analyzed by the CytoFLEX Flow Cytometer (Beckman Coulter, Inc., Brea, CA, USA).

### 4.10. Western Blot Analysis

In the preparation of cell lysates, RIPA buffer (Tech & Innovation, Shijiazhuang, Hebei, China) was supplemented with a 0.5 mM EDTA solution and a protease and phosphatase inhibitor cocktail (Thermo Fisher Scientific, Waltham, MA, USA), and was then added to the cells. The Pierce™ BCA Protein Assay Kit (Thermo Fisher Scientific, Waltham, MA, USA) was used for measuring the protein concentration. SDS-PAGE was used to separate the protein, and then membranes and antibodies were added to probe the protein. The membrane was incubated with primary antibodies and followed by secondary antibodies. To measure protein signals, the ChemiDoc XRS+ imaging system and ImageLab software version 4.1 (Bio-Rad, Hercules, CA, USA) were used.

### 4.11. Statistical Analysis

For statistical analysis, IBM SPSS statistics software version 23.0 (SPSS, Inc., Chicago, IL, USA) was used. A one-way ANOVA and Duncan’s multiple range test were performed for comparison with controls, which declared the difference significant at *p* < 0.05.

## 5. Conclusions

Our results demonstrated that PPCRE possesses immune-enhancing activity, including the activation of macrophages by increasing NO and cytokine secretion levels. This alteration in secretion levels affected the expression of immune mediator genes and proteins, and activated phagocytosis by stimulating macrophages through the NF-κB and MAPK signaling pathways. Therefore, PPCRE, as a potential immune modulator, may be used in the development of immune-enhancing drugs.

## Data Availability

All data are available online. No unpublished data have been used in this paper.

## Supplementary Materials

The following supporting information can be downloaded at: https://www.mdpi.com/article/10.3390/ijms251910660/s1.
