# Supplementary material for: Immunostimulatory Activity of a Mixture of Platycodon grandiflorum, Pyrus serotine, Chaenomeles sinensis, and Raphanus sativus in RAW264.7 Macrophages
Source: Int J Mol Sci. 2024 Oct 3;25(19):10660. doi: 10.3390/ijms251910660 (PMC11476558; doi:10.3390/ijms251910660)
Supplement: Supplementary file 1 [file ijms-25-10660-s001.zip › ijms-3074520-supplementary.pdf]

Supplementary Materials and methods

Immunostimulatory Activity of a Mixture of *Platycodon grandiflorum*, *Pyrus serotine*, *Chaenomeles sinensis*, and *Raphanus sativus* in RAW264.7 Macrophages

Weerawan Rod-in¶, Minji Kim ¶, A-yeong Jang, Yu Suk Nam, Tae Young Yoo, and Woo Jung Park*

* Correspondence: pwj0505@gwnu.ac.kr; Tel.: +82-33-640-2857

1. Analysis of active compounds by Ultra-performance liquid chromatography–tandem mass spectrometry (UPLC–MS/MS)

1.1. Sample preparation

The freeze-dried PPCRE (40 mg) was dissolved in 18% acetonitrile and filtered through a 0.45 μm PVDF syringe filter (Whatman, USA). The sample was kept at -80 °C until use, and 5 μL of sample was injected into UPLC-MS/MS systems.

1.2. Standard preparation

10 mg each of Platycoside E and Platycodin D standard substances were weighed and dissolved in a 10 mL volumetric flask with 18% acetonitrile to prepare a stock solution with a concentration of 1,000 μg/mL. This stock solution was then diluted with 18% acetonitrile to obtain working solutions at concentrations of 0.78, 1.56, 3.13, 6.25, 12.5, 25, and 50 μg/mL. A calibration curve was constructed using these prepared standard solutions.

1.3. LC-MS/MS conditions

The samples were separated using a Capcell Pak C18 UG 120 column in UPLC-MS/MS mode (Table S1). Platycodin D and Platycoside E in the PPCRE were identified by comparing their retention times with those of the standards. Their concentrations were calculated using calibration curves based on the linear relationship between the standards' concentrations and peak areas. The mass spectral parameters for the four coccidiostats are listed in Table S2.

Table S1. UPLC-MS/MS condition for analysis of Platycodin D and Platycoside E

Parameter	Condition			
UPLC	Column	Capcell Pak C18 UG 120, (5.0 µm, 4.6 x 250 mm)		
	Column Temp	30 ℃		
	Mobile Phase (Gradient)	Time (min)	A[1)] (%)	B[2)] (%)
		0	82	18
		15	75	25
		30	70	30
		35	82	18
		40	82	18
	Flow rate	1.0 mL/min		
	Injection volume	5 µL		
MS/MS	Mod	ESI negative	Curtain gas	25 psi
	Gas 1	40 psi	Ion spray voltage	-4.5kV
	Gas 2	60 psi	CAD	6 eV

Note: [1)] 0.1% formic acid in DW, [2)] Acetonitrile

Table 2. The parameters of mass spectrometer for analyzing Platycodin D and Platycoside E

Compound	Q1	Q3	DP	EP	CE	CXP
Platycodin D1	1223.3	469.2	-126	-10.0	-81.0	-10.0
Platycodin D2	1223.3	681.5	-120	-10.0	-90.0	-10.0
Platycoside E1	1547.7	469.1	-149	-11.0	-95.0	-16.0
Platycoside E2	1547.7	1005.6	-194	-11.0	-93.0	-44.0

Note: First quadrupole (Q1), third quadrupole (Q3), de-clustering potential (DP), entrance potential (EP), collision energy (CE) and collision cell exit potential (CXP)

2. qRT-PCR Analysis

Table S3. Primer sequences used in real-time qPCR

Target genes	Accession numbers	Primer sequence	
IL-1β	NM_008361.4	Forward	5′-GGGCCTCAAAGGAAAGAATC-3′
		Reverse	5′-TACCAGTTGGGGAACTCTGC-3′
IL-4	NM_021283.2	Forward	5′-ACAGGAGAAGGGACGCCAT-3′
		Reverse	5′-GAAGCCCTACAGACGAGCTCA-3′
IL-6	NM_031168.2	Forward	5′-AGTTGCCTTCTTGGGACTGA-3′
		Reverse	5′-CAGAATTGCCATTGCACAAC-3′
TNF-α	D84199.2	Forward	5′-ATGAGCACAGAAAGCATGATC-3′
		Reverse	5′-TACAGGCTTGTCACTCGAATT-3′
iNOS	BC062378.1	Forward	5′-TTCCAGAATCCCTGGACAAG-3′
		Reverse	5′-TGGTCAAACTCTTGGGGTTC-3′
COX-2	NM_011198.4	Forward	5′-AGAAGGAAATGGCTGCAGAA-3′
		Reverse	5′-GCTCGGCTTCCAGTATTGAG-3′
β-actin	NM_007393.5	Forward	5′-CCACAGCTGAGAGGGAAATC-3′
		Reverse	5′-AAGGAAGGCTGGAAAAGAGC-3′
